# Pitavastatin induces apoptosis in oral squamous cell carcinoma through activation of FOXO3a

**DOI:** 10.1111/jcmm.15389

**Published:** 2020-05-14

**Authors:** Naeun Lee, Nirmala Tilija Pun, Won‐Jun Jang, Jung Woo Bae, Chul‐Ho Jeong

**Affiliations:** ^1^ College of Pharmacy Keimyung University Daegu South Korea

**Keywords:** AMPK, drug repositioning, FOXO3a, OSCC, statin

## Abstract

Statins are a class of lipid‐lowering drugs that have recently been used in drug repositioning in the treatment of human cancer. However, the underlying mechanism of statin‐induced cancer cell death has not been clearly defined. In the present study, we evaluated the anticancer effect of pitavastatin on oral squamous cell carcinoma (OSCC), SCC15 and SCC4 cells and found that FOXO3a might be a direct target in pitavastatin‐induced cancer cell death. Our data revealed that pitavastatin selectively suppressed cell viability and induced intrinsic apoptosis in a FOXO3a‐dependent manner in SCC15 cells while no effect was observed in SCC4 cells. Notably, treatment with pitavastatin in SCC15 cells induced the nuclear translocation of FOXO3a via dual regulation of two upstream kinases, AMPK and Akt, resulting in the up‐regulation of PUMA, a transcriptional target gene of FOXO3a. Furthermore, our data revealed that FOXO3a‐mediated PUMA induction plays a role in pitavastatin‐induced intrinsic apoptosis in SCC15 cells. Taken together, our findings suggest that pitavastatin activates the FOXO3a/PUMA apoptotic axis by regulation of nuclear translocation of FOXO3a via Akt/FOXO3a or AMPK/FOXO3a signalling. Therefore, these findings might help to elucidate the underlying mechanism of the anticancer effects of pitavastatin on OSCC.

## 1 INTRODUCTION

Head and neck cancer is the seventh most common malignant tumour in the world, with more than 5.5 million cases diagnosed and 379 000 deaths globally in 2015.^1^ Oral squamous cell carcinoma (OSCC) is the most common oral cancer, accounting for 80%‐90% of head and neck cancer.^2^ Although chemotherapy and targeted therapy for the treatment of OSCC has been developed rapidly in the past years, the overall 5‐year survival rate for OSCC patients still remains at 50% due to the metastases and/or second tumours arising in patients following treatment.^3^ Therefore, the development of a safe and effective treatment strategy for OSCC is urgently needed.

Drug repositioning has recently drawn considerable attention as an approach used to identify alternative therapeutic uses of compounds that have failed in the development phase or to expand the use of drugs that are already in use.^4^ This approach has been widely applied to determine primary treatments for rare tumours lacking effective treatments and/or to develop secondary treatments for recurrent diseases.^5^ Recently, non‐oncological drugs including thalidomide have been repurposed as anticancer drugs^4^ and related clinical trials are underway. Furthermore, various drugs such as oxycodone, enzalutamide, itraconazole, nelfinavir, disulfiram, digoxin and metformin, which were previously used for various purposes, have been repurposed as anticancer drugs, which are now being tested in clinical trials.^6^

Statins, also known as 3‐hydroxy‐3‐methylglutaryl‐CoA (HMG‐CoA) reductase inhibitors, are widely used to treat high‐cholesterol patients via inhibition of cholesterol biosynthesis. Recently, the clinical benefits of statins have been demonstrated in the treatment of cancers such as lung, prostate and colon cancers; therefore, statins have drawn interest as potent anticancer drugs.^7^, ^8^, ^9^ In patients with head and neck cancer plus hyperlipidaemia, the intake of statins demonstrated higher overall survival of 2 years compared to patients with hyperlipidaemia without statin intake.^10^ A previous cohort study also showed that the use of statins in patients with primary inflammatory breast cancer improved their disease‐free survival.^11^

The molecular mechanism underlying the anticancer effects of statins have been studied and known to be mediated via broad regulation of cell proliferation, cell cycle, apoptosis, migration, invasion and metastasis.^12^ In particular, statins inhibit ovarian cancer cell proliferation and induce apoptosis by reducing the enzymatic activity of HMG‐CoA reductase, which is an important enzyme required for cholesterol synthesis in the mevalonate pathway.^13^ In addition, statins activate AMPK, a cellular energy sensor that maintains metabolic homeostasis under stress conditions,^14^, ^15^ and the activation of which is involved in the induction of apoptosis and suppression of cell viability in cancer cells.^14^ Furthermore, statins deactivate the PI3K/Akt/mTOR, and MAPK/ERK signalling, thereby leading to apoptosis and suppression of breast cancer cell proliferation.^16^ Although statins show anticancer effect in many cancers, there has been still insufficient data to demonstrate the effects of statins on OSCC and further discovery of statin with higher potency and better toxicity profile is needed for the future clinical trial.

FOXO3a, a protein of the Forkhead box class O (FOXO) subfamily, is a transcription factor that regulates multiple physiological and pathological processes such as proliferation, cell cycle progression, cell survival, DNA damage and apoptosis.^17^ Kinases such as Akt, ERK, SGK and IKKβ facilitate the nuclear export of FOXO3a via phosphorylation, which leads to ubiquitination and subsequent proteasomal degradation of FOXO3a.^18^ However, phosphorylation of FOXO3a by AMPK and MST1 facilitates nuclear translocation, thereby mediating multiple cellular processes by inducing the transcription of its target genes.^19^ In particular, FOXO3a transcriptionally induces Bim, Fas ligand (FasL) and p53 up‐regulated modulator of apoptosis (PUMA), thereby leading to apoptotic processes.^20^, ^21^ Since FOXO3a induces genes relating to apoptosis, it has been regarded as a tumour suppressor, and the expression level of FOXO3a has been found to decrease in various malignant tumours. For example, low expression of FOXO3a appears to be correlated with the progression, occurrence and poor prognosis in patients with breast, gastric or ovarian cancer.^22^, ^23^ However, many studies have also revealed that overexpression of FOXO3a was associated with poor prognosis in breast cancer and glioblastoma.^24^, ^25^ Therefore, it is still controversial and requires further studies to reveal the exact role of FOXO3a in human cancers.

A recent study has shown that FOXO3a can be regulated by simvastatin, and FOXO3a expression levels are correlated with metastasis‐free survival in patients with breast cancer.^26^ However, the mechanism of how FOXO3a is regulated and its role in cancer cell progression by statins is still unclear. Therefore, present study was performed with the aim to evaluate the anticancer effect of statins and elucidate the molecular mechanism by which statins regulate FOXO3a in OSCCs.

## 2 MATERIALS AND METHODS

### 2.1 Chemicals and reagents

AICAR, compound C, eosin, haematoxylin, 4′,6‐diamidino‐2‐phenylindole (DAPI), dimethyl sulfoxide (DMSO) and crystal violet were obtained from Sigma‐Aldrich (St. Louis, MO, USA). Simvastatin was purchased from LKT Laboratories, Inc (St. Paul, MN, USA), and pitavastatin calcium was purchased from HL Genomics (Gyeonggi‐Do, Republic of Korea). LY294002 and antibodies against p‐Akt (Ser437), Akt, p‐AMPK (Tyr172), AMPK, p‐FOXO3a (Ser253, Ser413), FOXO3a, PUMA, Caspase‐3, Caspase‐9, PARP, E‐cadherin, N‐cadherin, vimentin, β‐catenin, ZEB1, Snail2 and Lamin B1 were purchased from Cell Signaling Technology (Beverly, MA, USA), and GAPDH was purchased from Santa Cruz Biotechnology (Santa Cruz, CA, USA). A secondary antibody conjugated to horseradish peroxidase was procured from Thermo Fisher Scientific (Rockford, IL, USA).

### 2.2 Cell lines and cell culture

Human OSCCs, SCC4 and SCC15, were obtained from American Type Culture Collection (Manassas, VA, USA) and maintained in Dulbecco's modified Eagle's medium/F12 (DMEM/F12=1:1) supplemented with 10% FBS (HyClone Laboratories, Logan, UT, USA) and 1% penicillin/streptomycin at 37°C in a humidified incubator with 5% CO_2_.

### 2.3 RNA interference

Double‐stranded siRNAs against human FOXO3a (NM_001455.3), Akt (NM_001014431.1), AMPK (NM_001355028.1), PUMA (NM_001127240.2) were synthesized by Bioneer (Daejeon, Republic of Korea). Negative control siRNA was obtained from Bioneer (Cat. No.: SN‐1003). The sequence for siRNA duplexes used in this study are as follows: AKT: forward, 5′‐ GAC AAC CGC CAU CCA GAC U‐3′; reverse, 5′‐ AGU CUG GAU GGC GGU UGU C‐3′; AMPK: forward, 5′‐ CUG AGU UGC AUA UAC UGU A‐3′; reverse, 5′‐ UAC AGU AUA UGC AAC UCA G‐3′; FOXO3a: forward, 5′‐ CUA UCA UAU GGC AUU CUU A‐3′; reverse, 5′‐ UAA GAA UGC CAU AUG AUA G‐3′; PUMA: forward, 5′‐ GUA GAU ACC GGA AUG AAU U‐3′; reverse, 5′‐ AAU UCA UUC CGG UAU CUA C‐3′. SCC15 cells were seeded at 1.5 × 10^3^ cells/well in 96‐well microplates or 1 × 10^6^ cells/well in 100 mm dishes. After overnight incubation, the cells were transfected with small interfering RNA (siRNA) of target genes or with control siRNA using Oligofectamine transfection reagent (Invitrogen, Carlsbad, CA, USA) according to the supplier's protocol, followed by drug treatment. Gene silencing efficacy of siRNA was measured by Western blotting.

### 2.4 Caspase‐3/7 activity assay

Caspase‐3/7 activity was assessed using the Caspase‐Glo 3/7 assay kit (Promega Corporation, Madison, WI, USA) according to the manufacturer's instructions. In brief, cells were seeded at 1.5 × 10^3^ cells/well in 96‐well microplates and incubated overnight. At the end of the drug treatment, cells were incubated with a luminogenic substrate, and caspase‐3/7 activity was determined by measuring the luminescence that was generated from the cleavage of luminogenic substrate Z‐DEVD‐NH in presence of caspase‐3/7 protease using a micro‐plate reader.

### 2.5 Western blot analysis

Cell lines were distributed at 1 × 10^6^ cells/100 mm dish. After appropriate treatments, total cellular extracts were prepared using radio‐immunoprecipitation assay lysis buffer (RIPA) containing a Halt Protease Inhibitor Cocktail (Thermo Scientific). Next, 30‐40 µg of total proteins were loaded onto 6‐12% sodium dodecyl sulphate (SDS)‐polyacrylamide gel, separated by electrophoresis and transferred to PVDF membranes. Possible non‐specific binding was blocked by incubation with 5% BSA in TBS‐T for 1 hour and then incubated overnight at 4°C with specific primary antibodies. Membranes were then washed with TBS‐T four times, followed by incubation with secondary antibodies conjugated with horseradish peroxidase for 1 hour and washed with TBS‐T three times in 10 minutes intervals. Finally, images of proteins were captured using LAS‐3000 (Fuji, Tokyo, Japan).

### 2.6 Statistical analyses

Data were analysed by Student's *t* test or one‐/two‐way ANOVA using GraphPad Prism 5. All data are presented as mean ± SD *P* < 0.05 was considered statistically significant.

## 3 RESULTS

### 3.1 Pitavastatin inhibits metastatic properties in human oral squamous SCC15 cells

**Figure 1:**
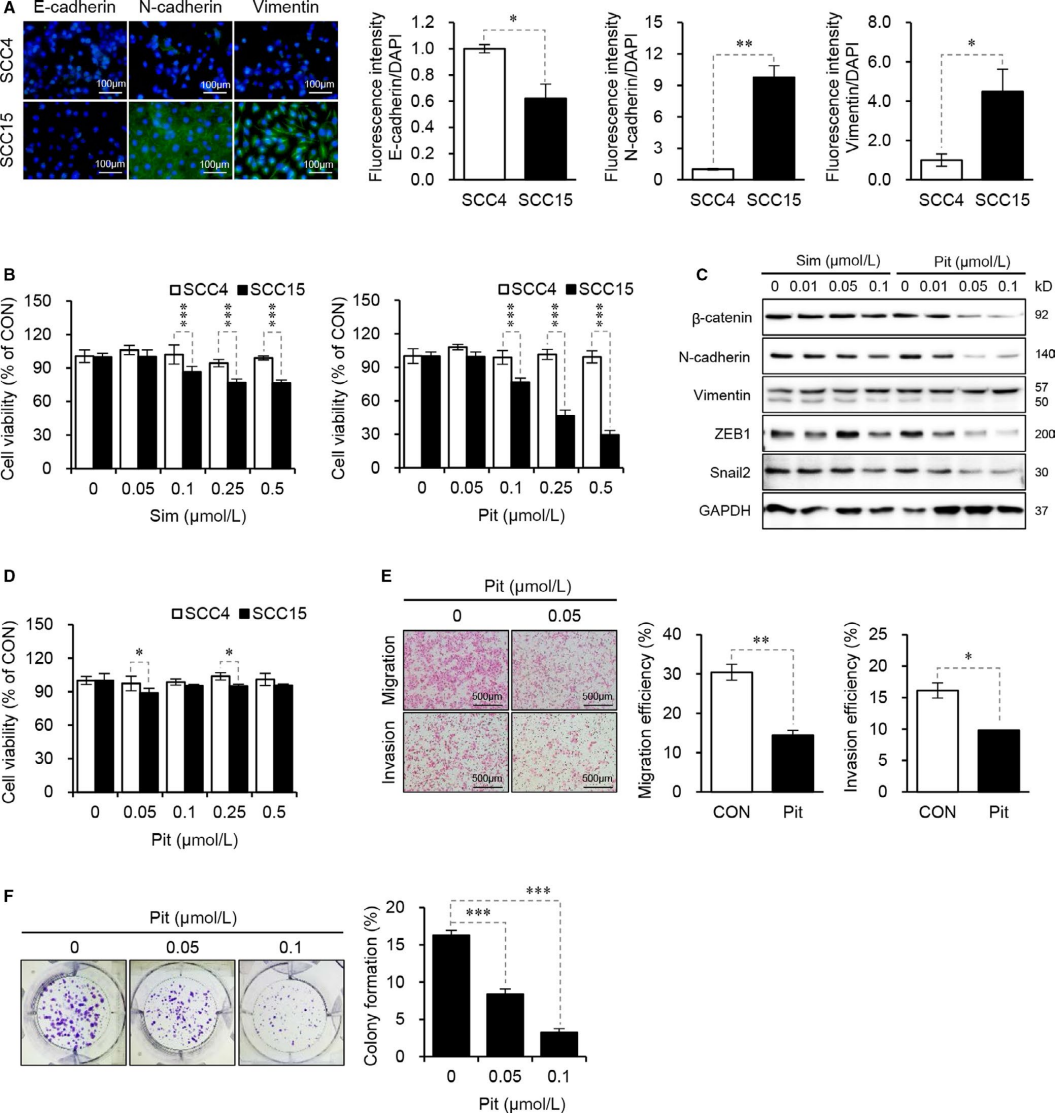
Pitavastatin inhibits cell viability and metastatic properties in human oral squamous SCC15 cells. A, Immunofluorescence staining of EMT markers in SCC4 and SCC15 cells. Cells were incubated with the indicated antibodies and stained with DAPI. Immunofluorescent cells were observed via fluorescence microscopy, and signal intensity was quantified and normalized to DAPI using the ImageJ software. Error bars represent mean ± SD (n = 3). Statistical significance was determined using Student's *t* test. *P < 0.05; **P < 0.01 vs SCC4 cells. B, Effects of statins on cell viability in SCC4 and SCC15 cells. Cells were seeded onto 96‐well plates (1.5 × 10^3^ cells/well) and treated with various concentrations of statins for 48 hours. Cell viability was measured using the CytoTox‐Glo cytotoxicity assay kit. Statistical analysis was performed by two‐way ANOVA. Error bars represent mean ± SD (n = 3). ***P < 0.001, compared to SCC4 cells. C, Effects of simvastatin and pitavastatin on the expression of EMT markers in SCC15 cells. SCC15 cells were treated with simvastatin or pitavastatin for 24 hours. Western blot analysis was performed to measure the protein expressions of EMT markers. GAPDH was used as an internal loading control. D, Short‐term effect of statins on cell viability in SCC4 and SCC15 cells. Cells were treated with statins for a short time duration (24 hours), and cell viability was measured using the CytoTox‐Glo cytotoxicity assay kit. Error bars show mean ± SD (n = 4). Statistical analysis was conducted using two‐way ANOVA. *P < 0.05, compared to SCC4 cells. E, Invasion and migration assay in SCC15 cells. Cells were plated onto Matrigel‐coated transwells (24‐well) and treated with pitavastatin for 24 hours. Live cells that invaded the lower surface were fixed, stained and manually counted using a light microscope. Statistical analysis was conducted using Student's *t* test. **P < 0.01, compared to control. Error bars show mean ± SD (n = 3). F, Colony formation assay. Cells were treated as indicated and incubated for 14 days, and colony formation was assessed by crystal violet staining. Shown is a representative image of three independent sets of experiments (left) and a quantification of colony formation (right). Statistical significance was determined using Student's *t* test, and error bars represent mean ± SD (n = 3). ***P < 0.001, compared to control

Our previous study revealed that SCC15 cells exhibit a typical mesenchymal phenotype and a higher dependency on aerobic glycolysis compared to other OSCC cell lines we tested.^27^, ^28^ Therefore, it is highly desirable to find an effective anticancer drug using this cell line model. In this study, we analysed the anticancer effect of statins, such as simvastatin and pitavastatin, on SCC4 and SCC15 human OSCC cells. To measure the expression pattern of epithelial‐mesenchymal transition (EMT) markers,^29^ cells were incubated with antibodies against E‐cadherin, N‐cadherin and vimentin and counter‐stained with DAPI. Immunofluorescent cells were then observed by fluorescence microscopy. As shown in Figure 1A, the expression of epithelial markers E‐cadherin was lower in SCC15 cells compared to SCC4 cells, whereas mesenchymal markers N‐cadherin and vimentin were highly expressed in SCC15 cells compared to SCC4 cells (Figure 1A), confirming the different epithelial and mesenchymal nature between two OSCC cell lines and this pattern is consistent with previous publication.^27^ As shown in Figure 1B, the treatment of both cells with pitavastatin and simvastatin for 48 hours significantly decreased cell viability in SCC15 cells but not in SCC4 cells. Among these statins, 0.5 µmol L^−1^ of pitavastatin inhibited cell viability by more than 50%, whereas simvastatin inhibited only about 10% at the same concentration in SCC15 (Figure 1B). Furthermore, the protein expression of mesenchymal markers such as β‐catenin, N‐cadherin, ZEB1 and snail2 was markedly decreased by treatment with pitavastatin in SCC15 cells, but not in SCC4 cells (Figure 1C). Based on these results, pitavastatin seems to be effective in SCC15 cells, and thus further experiments were conducted using pitavastatin. We also found that pitavastatin decreased the invasion and migration of SCC15 cells without affecting cell viability at 24 h (Figure 1D). As shown in Figure 1E, 0.05 µmol L^−1^ of pitavastatin decreased the migration and invasion by about 16% and 6.3%, respectively. Next, the effect of pitavastatin on the colony formation ability of SCC15 cells was measured. The results showed that colony formation was significantly suppressed by pitavastatin treatment (Figure 1F). Taken together, these results indicate that pitavastatin decreases cell viability and inhibits metastatic properties such as EMT, migration and invasion in SCC15 cells.

### 3.2 Pitavastatin selectively induces apoptosis in SCC15 cells

**Figure 2:**
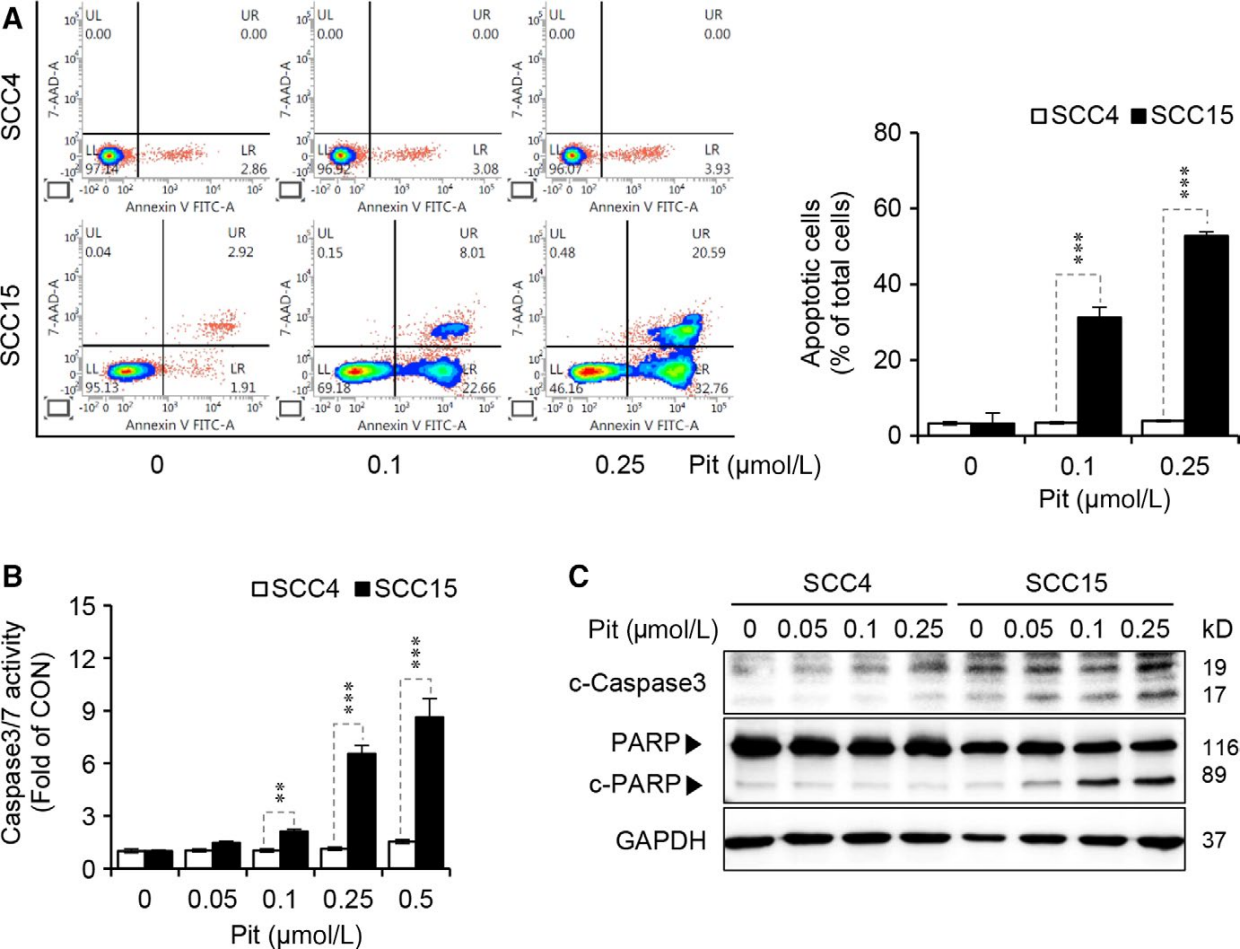
Pitavastatin selectively induces apoptosis in SCC15 cells. A, Cells were treated with pitavastatin for 48 hours, and the degree of apoptosis was measured by flow cytometric analysis with Annexin V staining (left), and the quantification of apoptosis is shown (right panel). Statistical analysis was conducted using two‐way ANOVA. Error bars represent mean ± SD (n = 3). ***P < 0.001 compared to SCC4 cells. B, After treatment with pitavastatin for 48 hours, caspase‐3/7 activity was measured using the Caspase‐3/7 Glo assay kit. Statistical analysis was conducted using two‐way ANOVA. Error bars represent mean ± SD (n = 4). **P < 0.01; ***P < 0.001 vs SCC4 cells. C, SCC4 and SCC15 cells were treated with pitavastatin for 24 hours, and the protein level of caspase‐3 and PARP were measured by Western blot analyses. GAPDH was used as a loading control

Next, we assessed the effect of pitavastatin on the induction of apoptosis by assessing for Annexin V‐positive cells via flow cytometry analysis. Our data revealed that pitavastatin did not induce apoptosis in SCC4 cells, whereas treatment with pitavastatin at a concentration of 0.1 µmol L^−1^ and 0.25 µmol L^−1^ increased apoptosis by 31% and 53%, respectively, in SCC15 cells (Figure 2A). Furthermore, pitavastatin‐induced caspase‐3/7 activity in SCC15 cells but not in SCC4 cells (Figure 2B), which was consistent with the results obtained from the flow cytometry analysis. The apoptotic effect of pitavastatin was further confirmed by Western blot analyses showing that the cleaved form of caspase‐3 and PARP were significantly increased by pitavastatin in a dose‐dependent manner (Figure 2C). These results altogether suggest that pitavastatin selectively induces apoptosis in SCC15 cells, but not in SCC4 cells.

### 3.3 Pitavastatin promotes translocation of FOXO3a by regulating AMPK and Akt signalling

**Figure 3:**
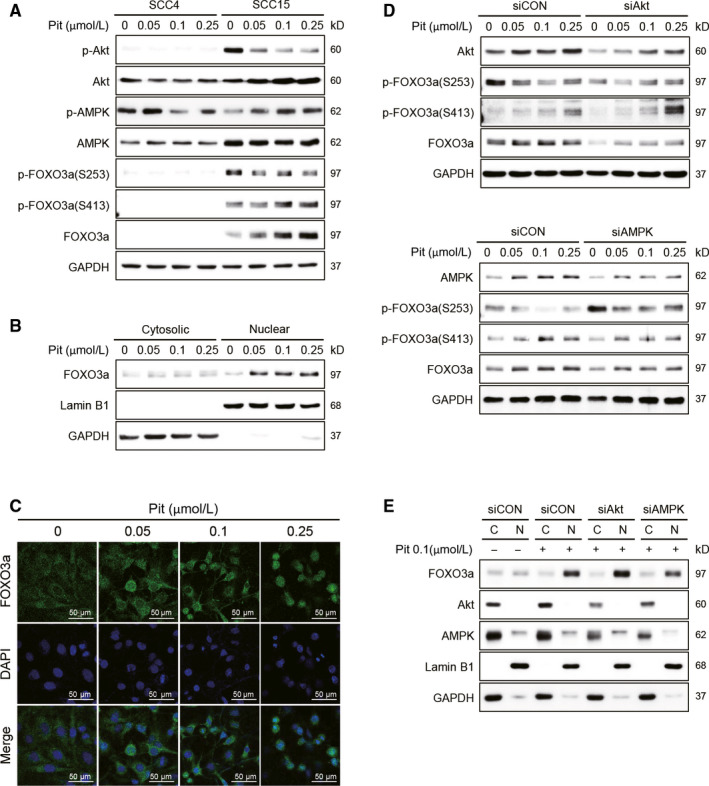
Pitavastatin promotes the translocation of FOXO3a by regulating Akt and AMPK phosphorylation. A, Cells were treated with pitavastatin for 24 hours, and the protein levels of Akt, AMPK and FOXO3a were measured by Western blot analyses. GAPDH was used as a loading control. B, After treatment of SCC15 cells with pitavastatin (24 hours), nuclear and cytoplasmic lysates were prepared, and the cytosolic and nuclear expressions of FOXO3a protein were analysed by Western blot analyses. GAPDH and Lamin B1 were used as cytoplasmic and nuclear loading controls, respectively. C, SCC15 cells cultured on sterile coverslips were treated with pitavastatin for 24 hours. Cells were then fixed with methanol, and FOXO3a was visualized by confocal microscopy after incubation with a rabbit polyclonal antibody against FOXO3a and stained with Alexa Fluor 488 (green)‐labelled anti‐rabbit antisera and DAPI. D, Cells were transfected with Akt or AMPK siRNA in the presence or absence of pitavastatin for 24 hours, and the expression of phosphorylated FOXO3a was measured by Western blot analyses. GAPDH was used as a loading control. E, Regulation of FOXO3a translocation by Akt or AMPK knockdown. After complete treatment, nuclear and cytoplasmic lysates were prepared, and the expression of nuclear and cytosolic FOXO3a proteins was detected by Western blotting

Simvastatin has been shown to induce apoptosis and inhibit EMT via suppression of PI3K/Akt signalling, thereby resulting in radiosensitivity in radioresistant oesophageal cancer cells.^16^, ^30^ In addition, other studies have shown that AMPK activation by lovastatin caused cytotoxicity and induced apoptosis of cancer cells such as OSCC and lung cancers.^31^, ^32^ Thus, we explored the possibility of whether Akt and AMPK signalling could be involved in pitavastatin‐mediated apoptosis in SCC15 cells. We have previously observed a higher level of phosphorylated‐Akt and lower level of phosphorylated‐AMPK in SCC15 cells compared to SCC4 cells.^28^ Since pitavastatin selectively showed anticancer effects only in SCC15 cells, we hypothesized that Akt and AMPK might be the possible regulatory proteins involved in the anticancer effects mediated by pitavastatin in SCC15 cells. Interestingly, no changes in the phosphorylation of Akt and AMPK were observed by treatment with pitavastatin in SCC4 cells, but the phosphorylated‐Akt level was decreased while the phosphorylated‐AMPK level was increased by pitavastatin in a dose‐dependent manner in SCC15 cells (Figure 3A). FOXO3a, a transcription factor regulating the transcription of diverse genes involved in apoptosis, has been known to be regulated by several upstream kinases including Akt and AMPK. Several reports have suggested that the phosphorylation of FOXO3a by Akt at serine 253 (S253) resulted in its export into the cytosol and subsequent inactivation,^33^ whereas AMPK phosphorylates FOXO3a at serine 413 (S413), thereby leading to nuclear translocation and ultimately induces its target genes to regulate cancer cell death.^34^ Therefore, we assessed the expression and phosphorylation of FOXO3a as a downstream signalling molecule of Akt and AMPK.^35^ Unlike SCC4 cells, which expresses low levels of FOXO3a, the already high basal levels of Foxo3a was increased after pitavastatin treatment in SCC15 cells, which was correlated with induction of apoptosis (Figure 3A). Interestingly, the phosphorylation of FOXO3a at S413 and S253 increased and decreased, respectively, by pitavastatin, which was consistent with the observed phosphorylated levels of AMPK and Akt (Figure 3A). Furthermore, the nuclear translocation of FOXO3a was examined by Western blotting and confocal microscopy. As shown in Figure 3B, the nuclear localization of FOXO3a was prominently increased by treatment with 0.05 µmol L^−1^ of pitavastatin. The nuclear translocation of FOXO3a was also confirmed by immunocytochemistry, where FOXO3a was condensed into the nucleus after pitavastatin treatment (Figure 3C). These results altogether confirmed that pitavastatin increased the activity of FOXO3a by increasing its phosphorylation and nuclear translocation. Next, we investigated the role of Akt and AMPK in the activation of FOXO3a by knockdown of their respective genes. As shown in Figure 3D (upper panel), the decrease in p‐FOXO3a (S253) as well as the increase in p‐FOXO3a (S413) by pitavastatin was further enhanced by gene silencing of Akt, respectively. The increase in p‐FOXO3a (S413) by gene silencing of Akt might have resulted from the inhibition of FOXO3a degradation because Akt‐mediated phosphorylation at S253 of FOXO protein facilities the binding by 14‐3‐3 protein or Skp2 leading to its degradation.^36^, ^37^ Opposite to the effect of Akt silencing, an increase in p‐FOXO3a (S253) and a decrease in p‐FOXO3a (S413) by pitavastatin were observed after AMPK gene silencing (Figure 3D, lower panel). Furthermore, modulation of FOXO3a activation by Akt and AMPK signalling was confirmed by measuring the amount of FOXO3a expression in the nucleus after gene silencing of Akt or AMPK in the presence or absence of pitavastatin. As shown in Figure 3E, nuclear expression of FOXO3a was increased by gene silencing of Akt, while decreased by gene silencing of AMPK, suggesting that Akt suppresses while AMPK induces FOXO3a activation in SCC15 cells.

### 3.4 Akt/AMPK signalling is involved in the induction of apoptosis by pitavastatin in SCC15 cells

**Figure 4:**
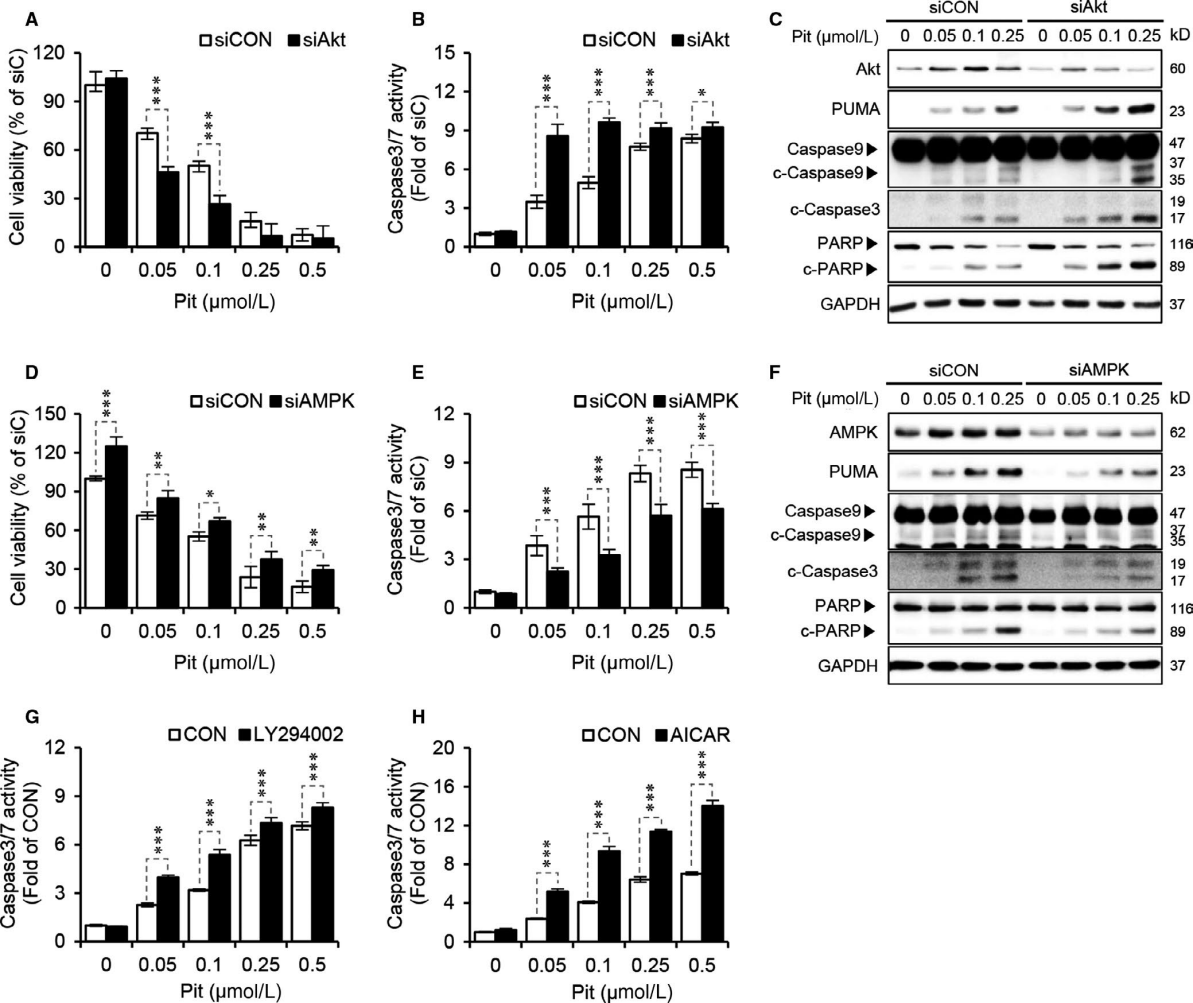
Pitavastatin promotes apoptosis by regulating Akt and AMPK signalling. A and B, SCC15 cells plated on 96‐well plates were transfected with Akt siRNA in the presence or absence of the indicated concentrations of pitavastatin for 48 hours. Cell viability was then measured by using the CytoTox‐Glo cytotoxicity assay (A), and caspase‐3/7 activity was measured using a Caspase‐3/7 assay kit (B). Statistical analysis was performed using two‐way ANOVA. Error bars represent mean ± SD (n = 4). *P < 0.05; ***P < 0.001 vs siCON. (C) Cells were transfected with Akt siRNA in the presence or absence of pitavastatin and then incubated for 24 hours, and the protein levels of apoptotic markers were measured by Western blot analyses. GAPDH was used as a loading control. D, E, Cell viability or caspase‐3/7 activity by pitavastatin in AMPK knockdown cells. After transfection of cells with AMPK siRNA, followed by pitavastatin treatment for 48 hours, cell viability was measured using the CytoTox‐Glo cytotoxicity assay (D), and caspase‐3/7 activity was measured using a Caspase‐3/7 assay kit (E). Statistical analysis was done by two‐way ANOVA. Error bars represent mean ± SD (n = 4). **P < 0.01; ***P < 0.001 vs siCON. (F) After AMPK siRNA and pitavastatin treatment for 24 hours, protein levels of apoptotic markers were measured by Western blot analyses. GAPDH was used as a loading control. (G, H) Modulation of caspase‐3/7 activity by LY294002 or AICAR in pitavastatin‐treated SCC15 cells. Cells were treated with 10 µmol L^−1^ LY294002 (G) or 100 µmol L^−1^ AICAR (H) in the presence or absence of the indicated concentrations of pitavastatin for 48 hours, and caspase‐3/7 activity was measured by Caspase‐3/7 activity assay kit. Statistical analysis was performed by two‐way ANOVA. Error bars represent mean ± SD (n = 4). ***P < 0.001 vs CON

As apoptosis is regarded as a major determinant of cell viability, we checked if pitavastatin‐mediated suppression of cell viability and induction of apoptosis might be the results of dual regulation of Akt and AMPK. The suppression of cell viability (Figure 4A) and activation of caspase‐3/7 (Figure 4B) by pitavastatin were further enhanced by siAkt compared with siCON. Furthermore, as shown in Figure 4C, induction of all the apoptotic markers such as PUMA, cleaved caspase‐3/9 and cleaved‐PARP by pitavastatin was further enhanced by siAkt. The role of Akt in apoptosis was further confirmed by measuring caspase‐3/7 activity by using the well‐known PI3K inhibitor LY294002, where an increase in caspase‐3/7 activity by pitavastatin was further enhanced by LY294002 pre‐treatment (Figure 4G), consistent with the result obtained by gene silencing of Akt (Figure 4B). These results suggest that Akt negatively regulates the apoptotic process, and suppression of Akt signalling could be a promising strategy for the treatment of oral cancer cells by pitavastatin. In addition, gene silencing of AMPK significantly abrogated the suppression of cell viability (Figure 4D) and inhibited the activation of caspase‐3/7 (Figure 4E) by pitavastatin. Similarly, the expression of apoptotic markers by pitavastatin was significantly suppressed by AMPK gene knockdown (Figure 4F). A similar result was obtained by using the well‐known AMPK activator AICAR (Figure 4H). These results further confirm that AMPK is involved in the suppression of cell viability and induction of apoptosis by pitavastatin in SCC15 cells.

### 3.5 Pitavastatin induces apoptosis through FOXO3a/PUMA signalling

**Figure 5:**
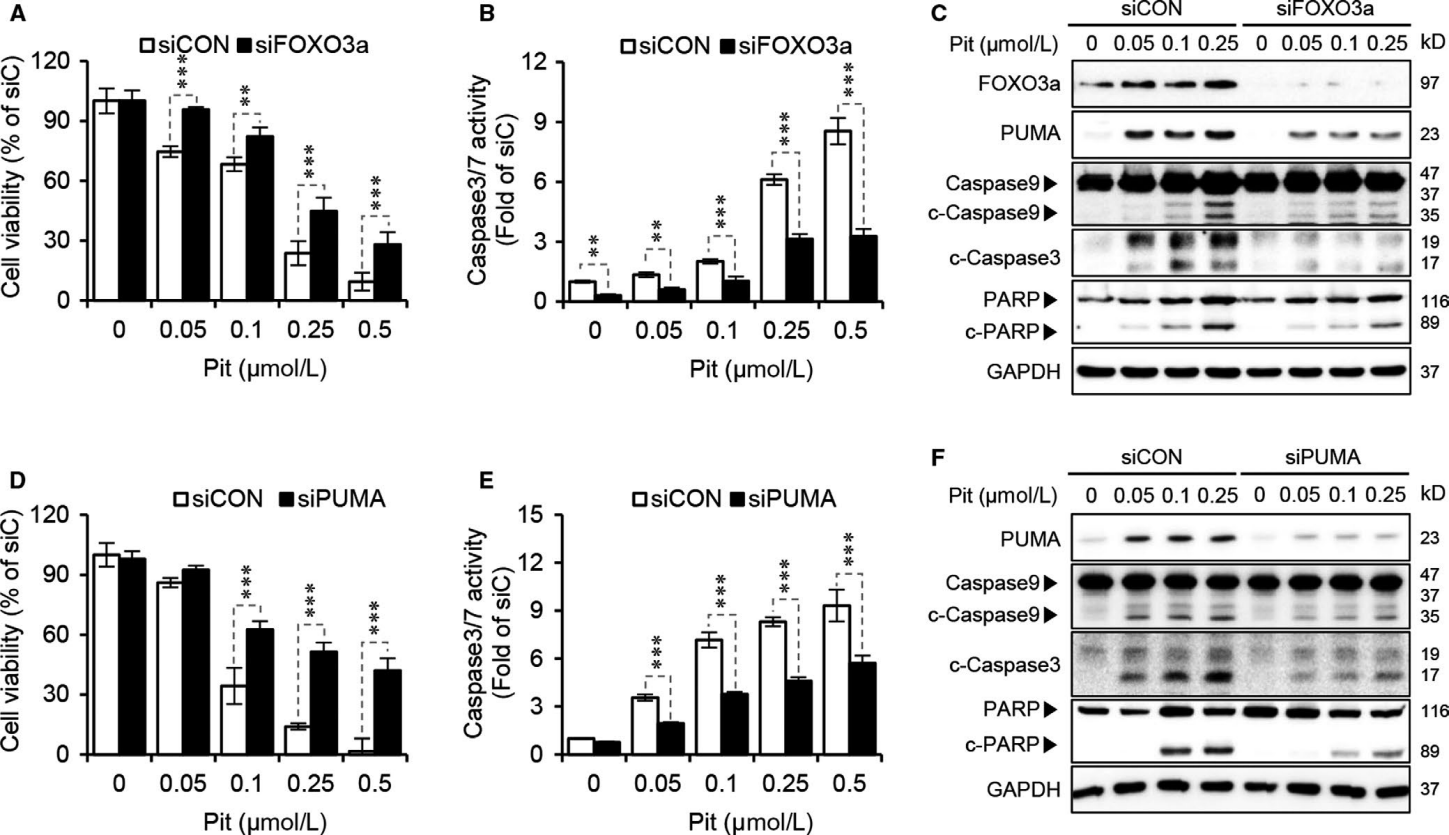
Pitavastatin induces apoptosis through FOXO3a/PUMA signalling. A, B, SCC15 cells plated on 96‐well plates were transfected with FOXO3a siRNA in the presence or absence of the indicated concentrations of pitavastatin and then incubated for 48 hours. Cell viability was then measured using the CytoTox‐Glo cytotoxicity assay (A), and caspase‐3/7 activity was measured by using a Caspase‐3/7 assay kit (B). Statistical analysis was performed by two‐way ANOVA. Error bars represent mean ± SD (n = 4). **P < 0.01; ***P < 0.001 vs siCON. (C) Cells were transfected with FOXO3a siRNA in the presence or absence of pitavastatin for 24 hours, and protein levels of apoptotic markers were measured by Western blot analyses. (D and E) Cell viability or caspase‐3/7 activity by pitavastatin in PUMA knockdown cells. Cells were transfected with PUMA siRNA in the presence or absence of pitavastatin for 48 hours. Cell viability was measured by CytoTox‐Glo cytotoxicity assay (D), and caspase‐3/7 activity was measured by the Caspase‐3/7 assay kit (E). Statistical analysis was conducted using two‐way ANOVA. Error bars represent mean ± SD (n = 4). ***P < 0.001 vs siCON. (F) After treatment with PUMA siRNA and pitavastatin for 24 hours, protein levels of apoptotic markers were measured by Western blot analyses. GAPDH was used as a loading control

Next, we determined whether FOXO3a regulated by Akt and AMPK could modulate apoptosis in SCC15 cells. Our data revealed that the suppression of cell viability and activation of caspase‐3/7 by pitavastatin were abrogated by gene silencing of FOXO3a (Figure 5A,B). As we expected, the expression level of apoptotic markers was lower in the siFOXO3a group than in the siCON group (Figure 5C). These results strongly suggest that FOXO3a is involved in the pitavastatin‐mediated apoptotic process in SCC15 cells. FOXO3a is a transcription factor that can regulate both extrinsic and intrinsic apoptosis pathway especially by transcribing FasL (extrinsic) and Bim or PUMA (intrinsic).^20^, ^21^, ^33^ Statins have been shown to induce apoptosis via down‐regulation of anti‐apoptotic protein Bcl‐2 rather than up‐regulation of FasL or p53, supporting the notion that statins can mediate apoptosis via the intrinsic pathway.^38^ When we measured the expression of Bim and PUMA after the pitavastatin treatment, which are the proteins involved in the intrinsic apoptotic pathway, PUMA was found to be increased by pitavastatin in a dose‐dependent manner while Bim was not changed (data not shown). Thus, PUMA was selected as a target gene of FOXO3a involved in the induction of intrinsic apoptosis. Like FOXO3a, gene silencing of PUMA significantly abrogated the suppression of cell viability and inhibited the activation of caspase‐3/7 by pitavastatin (Figure 5D,E). Consistently, knockdown of PUMA resulted in a significant decrease in pitavastatin‐induced expression of apoptotic markers (Figure 5F). Taken together, these results confirm that FOXO3a‐mediated PUMA induction was dominantly involved in a reduction of cell viability and induction of apoptosis by pitavastatin in SCC15 cells.

## 4 DISCUSSION

Recent drug repositioning studies have revealed the anticancer effect of statins in different types of cancer. Moreover, many studies have been conducted to elucidate their mechanism of action in various cancer cells. The main anticancer mechanism of statins is the inhibition of the mevalonate pathway, which is essential in the maintenance of cellular functions such as cell membrane integrity, cell cycle progression and protein synthesis and the inhibition of which leads to the suppression of cancer growth and progression.^39^ Another report indicates that the treatment of metastatic mouse melanoma cells with simvastatin and fluvastatin inhibits cell growth and metastasis via suppression of matrix metalloproteinases (MMPs), very late antigens (VLAs) and Rho prenylation, which are crucial for cell cycle progression and cell motility. In this experimental model, the cell cycle arrest was enhanced via the up‐regulation of cell cycle regulators such as p53, p27 and p21.^40^ Fluvastatin induces apoptosis of HSC‐3, an OSCC cells and that was found to be mediated via the inhibition of HMG‐CoA reductase leading to suppression of geranylgeranyl pyrophosphate (GGPP) biosynthesis; a substrate of the mevalonate pathway, and ERK1/2 phosphorylation.^41^ Also, simvastatin induces apoptosis in a mouse model of osteosarcoma via activation of AMPK/p38MAPK signalling, which is dependent on the inhibition of the mevalonate pathway.^14^ A recently developed statin, pitavastatin,^42^ exhibits anticancer effect synergistically with prednisolone or with pictilisib in ovarian cancer cells.^43^, ^44^

In the present study, the anticancer effect of statin, particularly pitavastatin was confirmed in SCC15 cells while almost no effect was observed in SCC4 cells. This can imply that the higher selectivity of pitavastatin in SCC15 between two OSCC might be related in part to the higher aggressiveness, invasiveness and metastatic nature of SCC15 compared to SCC4. Consistent with our previous data,^27^ we observed the low expression of E‐cadherin and high expression of N‐cadherin and vimentin in SCC15 cells compared to SCC4 cells (Figure 1A). The higher expression of N‐cadherin and vimentin denotes that SCC15 cells are more invasive and metastatic than SCC4 cells. Interestingly, our data indicated that pitavastatin shows a better potency in SCC15 cells compared to SCC4 cells. One possibility of differential sensitivity of SCC15 towards statin might be due to the higher expression of FOXO3a and its upstream kinases such as Akt and AMPK in SCC15 cells. The different patterns of Akt and AMPK modulation by pitavastatin (Figure 3A) might be one of the reasons. It is well known that activation of Akt is important in cancer cell proliferation and progression,^45^ whereas activation of AMPK is important in the suppression of tumour progression.^46^ Pitavastatin did not induce the activation of Akt, AMPK and FOXO3a in SCC4 cells but it decreased Akt phosphorylation while increased AMPK and FOXO3a phosphorylation as well as FOXO3a expression in SCC15 cells (Figure 3A). Our data demonstrated that the high basal expression of FOXO3a was maintained and, increased by the treatment with pitavastatin in SCC15 cells, but not in SCC4 cells. It is noteworthy that the differential activation of Akt and AMPK between the two OSCCs by pitavastatin might be crucial in the regulation of FOXO3a.

The protein activity and biological role of FOXO3a are determined by post‐translational modifications, including phosphorylation, acetylation and ubiquitination.^47^ AMPK phosphorylates FOXO3a at six positions (Thr179, Ser399, Ser413, Ser439, Ser555 and Ser588), which then leads to the transcription of various genes.^34^ AMPK‐mediated FOXO3a activation has been shown to induce cell cycle arrest and apoptosis in breast cancer cells and hepatoma cancer cells.^48^, ^49^ FOXO3a activation induced by the inhibition of PI3K/Akt is involved in cisplatin‐induced cytotoxicity in several cancers such as colorectal, lung and OSCC, suggesting that drugs that deactivate PI3K/Akt could be a promising anticancer therapy.^50^, ^51^ In the present study, our data also revealed that the suppression of cell viability and activation of caspase‐3/7 by pitavastatin was additively enhanced by silencing of Akt (Figure 4A‐C). According to Figure 3D, the decrease in p‐FOXO3a (S253) and increase in p‐FOXO3a (S413) by pitavastatin was further enhanced by the silencing of Akt (Figure 3D). Akt signalling is well known to mediate cancer cell proliferation and tumour growth. For example, HBP1 induction by ROS scavenger, NAC, decreases cell viability and induces apoptosis in oral cancer cells and this effect was mediated by inhibition of Akt activation.^52^ Further, induction of cell death by an anti‐oxidant, quercetin, is mediated by inhibition of PI3K/Akt/mTOR, Wnt/β‐catenin and STAT pathways.^53^ Furthermore, miRNA‐21 (miR‐21) inhibits PTEN/Akt/GSK3 signalling axis to inhibit lung cancer cell proliferation and EMT.^54^ In our experimental model as well, gene silencing of Akt further enhanced the suppression of cell viability and activation of caspase‐3/7 by pitavastatin and it shows that pitavastatin and Akt gene knockdown act to an additive mechanism. Thus, it can be assumed that the additive mechanism by gene silencing of Akt in pitavastatin‐treated condition might be due to the inhibition of other signalling pathways such as Akt/HBP1, PI3K/Akt/mTOR and Akt/GSK3 in addition to Akt‐FOXO3a signalling. Therefore, we reasoned that the (a) inhibition of Akt activation by pitavastatin seems to be incomplete and (b) other pathways as well as Akt‐FOXO3a can be contributed to an additive mechanism by pitavastatin and gene silencing of Akt.

**Figure 6:**
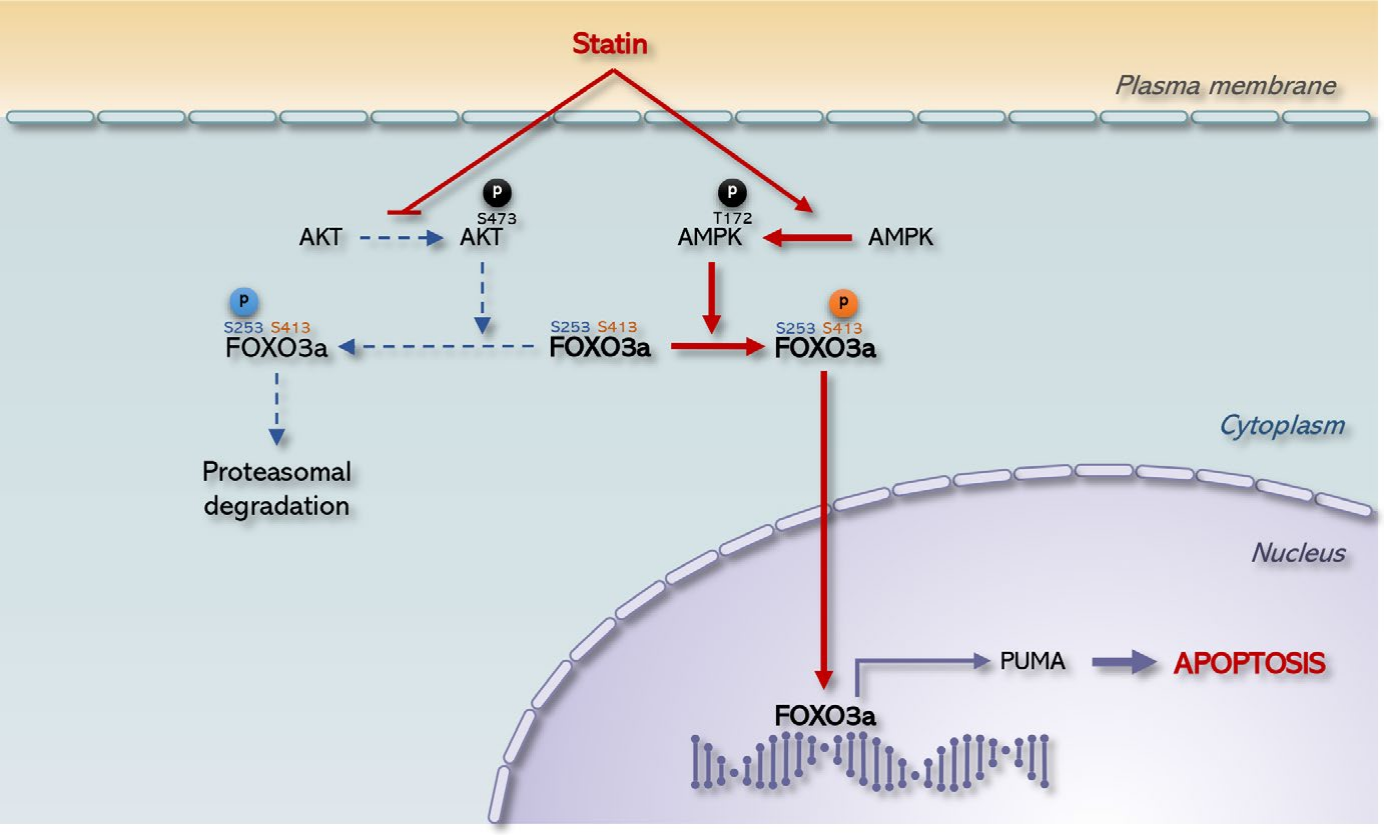
Proposed model of the anticancer effect of statin in SCC15 cells. Akt normally phosphorylates FOXO3a at S253, which then leads to ubiquitination and finally proteasomal degradation. However, AMPK phosphorylates FOXO3a at S413, resulting in FOXO3a activation and nuclear translocation. Statins decrease Akt phosphorylation while increasing AMPK phosphorylation, and both of these mechanisms promote FOXO3a nuclear translocation, which results in the downstream transcription of PUMA, an apoptotic protein that induces apoptosis in SCC15 cells

Our data suggest a molecular mechanism by which statins induces anticancer activity by modulating FOXO3a. Pitavastatin induces nuclear translocation of FOXO3a by inhibiting Akt and activating AMPK, which results in the induction of PUMA, causing cancer cell death (Figure 6). It has been accepted that the activation of FOXO3a inhibits the propagation, tumour formation and invasive nature of cancer, while inhibition of its activation induces tumour formation. Many anticancer drugs have been found to target FOXO3a protein. For example, paclitaxel and KP372‐1 (a multiple kinase inhibitor) activate FOXO3a by reducing Akt activation, thereby leading to cancer apoptosis.^55^, ^56^ Also, drugs such as trastuzumab and cetuximab activate FOXO3a to induce apoptosis in mouse melanoma models.^40^ Consistent with this, in this study, we also demonstrated that the Akt/FOXO3a and AMPK/FOXO3a axes are directly involved in statin‐induced apoptosis in OSCCs.

Apoptosis is normally triggered by two main pathways: extrinsic, which is mediated by the activation of death receptors (Fas and TNFR) and subsequent activation of caspase‐8, and intrinsic, which is mediated by the mitochondria and subsequent activation of caspase‐9. Both the extrinsic and intrinsic pathways result in PARP activation, thereby leading to cell death. Of note, FOXO3a has been known to induce both apoptotic pathways.^57^ It has been shown that FOXO3a mediates extrinsic apoptosis via transcriptionally up‐regulating FasL^33^ and induces intrinsic apoptosis by increasing the expression of proapoptotic Bcl‐2 family members such as Bim and PUMA.^20^, ^58^ These proteins activate caspase‐9, leading to the cleavage of caspase‐3/7 and subsequent widespread cell death. A previous study has observed that apoptosis induced by atorvastatin and fluvastatin was found to be dependent on the regulation of anti‐apoptotic protein Bcl‐2 rather than up‐regulation of FasL or p53.^38^ Our study also revealed that pitavastatin induces oral cancer cell apoptosis via FOXO3a‐mediated up‐regulation of PUMA, which leads to the activation of caspase‐3/9 (Figure 5), suggesting that the intrinsic apoptosis pathway is regulated by statins.

Collectively, by investigating the anticancer activity of statin in OSCCs, it was confirmed that the transcriptional activity of FOXO3a is increased by the regulation of Akt and AMPK activity, which leads to the induction of intrinsic apoptosis in OSCC. Therefore, we propose that statins can be used as anticancer agents, and the effect of statin is mainly due to the Akt/FOXO3a or AMPK/FOXO3a axis.

## CONFLICT OF INTEREST

The authors declare no conflicts of interest.

## Data Availability

The data that support the findings of this study are available from the corresponding author upon reasonable request.
